# Laser irradiated fluorescent perfluorocarbon microparticles in 2-D and 3-D breast cancer cell models

**DOI:** 10.1038/srep43408

**Published:** 2017-03-06

**Authors:** Chengcheng Niu, Long Wang, Zhigang Wang, Yan Xu, Yihe Hu, Qinghai Peng

**Affiliations:** 1 Department of Ultrasound Diagnosis, The Second Xiangya Hospital, Central South University, Changsha, Hunan 410011, China; 2 Department of Orthopedics, Xiangya Hospital, Central South University, Changsha, Hunan 410008, China; 3 Second Affiliated Hospital, Institute of Ultrasound Imaging, Chongqing Medical University, Chongqing, 400010, China

## Abstract

Perfluorocarbon (PFC) droplets were studied as new generation ultrasound contrast agents via acoustic or optical droplet vaporization (ADV or ODV). Little is known about the ODV irradiated vaporization mechanisms of PFC-microparticle complexs and the stability of the new bubbles produced. In this study, fluorescent perfluorohexane (PFH) poly(lactic-co-glycolic acid) (PLGA) particles were used as a model to study the process of particle vaporization and bubble stability following excitation in two-dimensional (2-D) and three-dimensional (3-D) cell models. We observed localization of the fluorescent agent on the microparticle coating material initially and after vaporization under fluorescence microscopy. Furthermore, the stability and growth dynamics of the newly created bubbles were observed for 11 min following vaporization. The particles were co-cultured with 2-D cells to form 3-D spheroids and could be vaporized even when encapsulated within the spheroids via laser irradiation, which provides an effective basis for further work.

Current tumor chemotherapy is associated with severe undesirable effects on non-target organs and tissues. In addition, anomalous tumor vascularization and reduced lymphatic drainage in tumor tissue results in increased interstitial fluid pressure, which hinders the conventional transport of drug carriers across blood vessel walls, which in turn can lead to the survival of some cancer cells^1^,^2^. Thus, the efficient delivery of drugs to target tumor tissues is critical to reduce unintended and undesirable toxicity to healthy cells, tissues and organs^3^.

Hyperthermia treatments for cancer, through the localized application of thermal energy to cause protein denaturation and coagulation necrosis, have generated intensive research interest^4^,^5^. However, commonly used hyperthermia processes are still controversial in terms of disease-free survival, clinical efficacy, and long-term local recurrence rates.

We propose a new therapeutic approach by combining targeted physical destruction, such as laser ablation, and chemical therapy, such as targeted drug delivery, to maximize the curative effect and reduce non-target tissue damage. High optical absorption is an essential property for a drug carrier in effective photothermal and drug therapy and the method proposed herein is underpinned by the following caveats: upon the incorporation of a photo-absorbing agent, the laser energy absorbs efficiently and converts into heat for the specific ablation of cancer cells and the drug in the carrier should be simultaneously released to enhance the therapeutic effect at the tumor margin.

Recent studies have demonstrated that active ultrasound sonication or laser irradiation can vaporize perfluorocarbon (PFC) droplets into gas bubbles via acoustic or optical droplet vaporization (ADV or ODV)^6^,^7^,^8^,^9^. These bubbles can be used as ultrasound contrast agents^10^,^11^ for cancer therapy, via vessel occlusion^12^,^13^,^14^, and thermal ablation^15^,^16^, to liberate the encapsulated chemotherapeutic agents to a target region for ultrasound-mediated drug or gene delivery^7^,^17^,^18^,^19^. Liquid PFCs have negligible absorption in the visible/infrared region of the spectrum. Therefore, an optically absorbent material, such as a fluorescent dye, needs to be incorporated into the PFC particles to facilitate vaporization upon irradiation with an appropriate laser^20^,^21^,^22^.

Upon exposure to laser irradiation, the phase-shift particles absorb the laser energy and transform it into heat energy, which facilitates vaporization of the PFCs inner liquid. The phase transformation from liquid to gas generates large numbers of micron-sized bubbles, which generates heat energy and leads to thermoelastic expansion that synergistically enhances the effect of thermal ablation on local tumors. Moreover, the microbubbles generated can be used as ultrasound contrast agents, and the particle-encapsulated drugs are released to tissues through phase conversion progress and bubble oscillation^1^,^7^,^18^,^23^,^24^.

Prior to exploring the effective delivery of therapeutic agents in human clinical trial settings, thorough *in vitro* testing of candidate drugs and their delivery mechanism is required to determine critical parameters such as effectiveness, toxicity and safety^3^. Typically, two-dimensional (2-D) cell culture is used to evaluate drug delivery mechanisms against target cells. However, the associated lack of appropriate physiological barriers that fail to reproduce the anatomy or physiology of a tissue limits the quantity and quality of useful informative data^25^,^26^,^27^. To overcome this limitation, *in vitro* models of tissues, such as three-dimensional (3-D) multicellular spheroids, have been designed to produce enhanced models for better prediction of drug effects and delivery mechanisms, compared to conventional 2-D culture^28^,^29^,^30^,^31^. Spheroids, formed by self-assembled cell clusters, are 3-D models of solid tumors^32^. Large spheroids can develop central necrosis and regions of hypoxia characteristic of many cancers, which is critical for the effective testing of anti-cancer therapeutics^33^,^34^,^35^.

This study investigated fluorescently labelled perfluorohexane (PFH) poly (lactic-co-glycolic acid) (PLGA) particles as a model to study the process of particle vaporization and bubble stability following vaporization in 2-D and 3-D cell models. DiI dye was used as a drug model to observe release after vaporization. Additionally, the dye, which was encapsulated in the shell of the particles, acts as a good optical adsorbent. The particles were first co-cultured with cells to form 3-D spheroids. The particles were then vaporized within the spheroids via laser irradiation, which is a feasible method used for targeted drug delivery and the thermal ablation of tissues and organs *in vivo*^1^,^2^. Upon vaporization via laser irradiation, the particles transform into bubbles with the delivery of a dye payload. We observed the localization of the fluorescent agent on the coating material of the microparticles before and after vaporization under fluorescent microscopy. Furthermore, the stability and growth dynamics of the newly created bubbles were observed for 11 min following vaporization.

## Results

### Characterization of fluorescent microparticles

**Figure 1:**
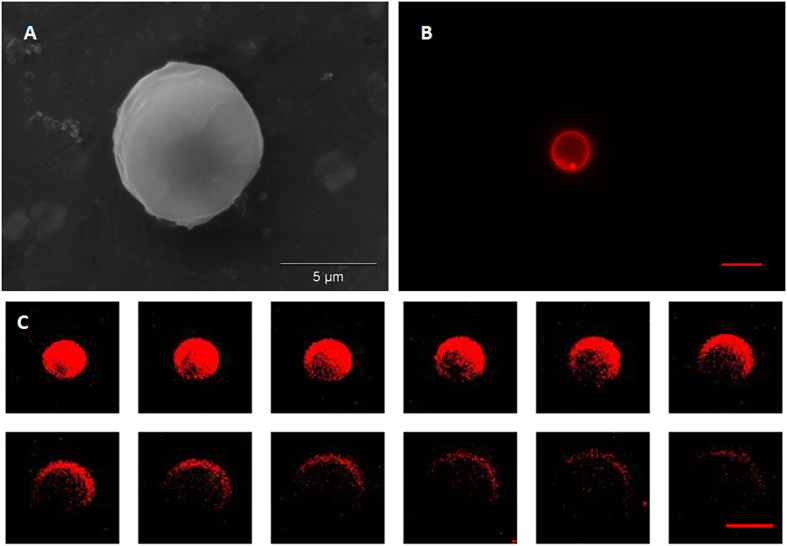
SEM, fluorescent microscopy, and confocal microscopy images of a DiI-labelled microparticle. (**A**) SEM image; (**B**) Fluorescent microscopy image; (**C**) Confocal microscopy images of a DiI-labelled microparticle from the surface plane to the inner planes. Scale bars represent 5 μm.

A representative SEM image, Fig. 1A, shows that the fluorescent shell PFC microparticles exhibited a smooth spherical morphology. In this study, all of the suspended microparticles ranged in size from 3–10 μm. However, only microparticles with a diameter of 5–8 μm were used for further experimentation. The fluorescence microscopy image of the fluorescent shell PFC microparticle in Fig. 1B shows a distinct fluorescent circular rim pattern on one plane, which suggests that the fluorescence marker selectively labelled the PLGA shell of the microparticles, avoiding the PFC liquid cores. The confocal microscopy images shown in Fig. 1C further demonstrates that PFC liquid was successfully loaded in the PLGA microparticles with no fluorescence signal.

**Figure 2:**
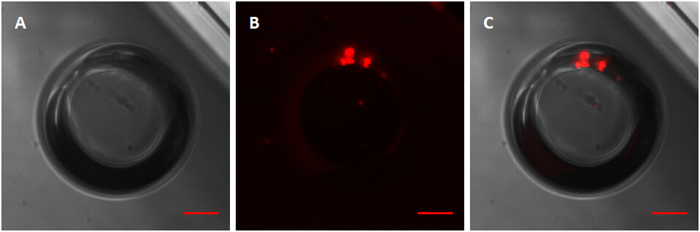
Brightfield and fluorescent microscopy images of a vaporized DiI-labelled bubble. (**A**) Brightfield image; (**B**) Fluorescent microscopy image; (**C**) Merged picture of (**A**,**B**). Scale bars represent 10 μm.

Additionally, fluorescence microscopy imaging of vaporized bubbles was undertaken to decipher if and where the bubbles emitted a fluorescence signal. Figure 2 shows that after vaporization the fluorescence signal only existed as a small point on the PLGA assembled edge of the bubble, while no fluorescence was visible within the newly created PFH bubble.

**Figure 3:**
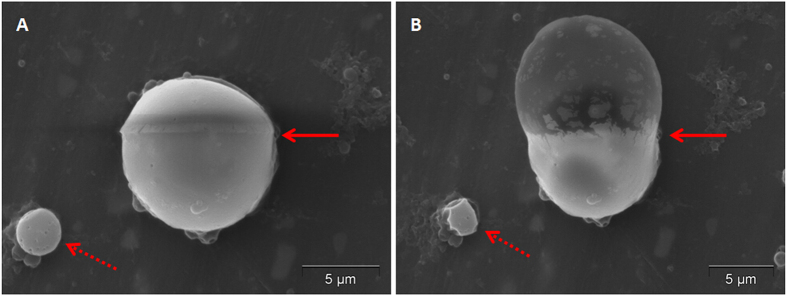
SEM images of a DiI-labelled microparticle before and after vaporization. (**A**) Before vaporization; (**B**) After vaporization. Arrows points to the area of the microparticle where vaporization occurred under the influence of the high electron beam.

Interestingly, when we performed SEM, some of the microparticles vaporized under the high power electron beam irradiation. Figure 3 shows a microparticle before and after SEM induced vaporization. The upper portion of the microparticle was initially disturbed under SEM conditions (Fig. 3A), which evolved to a balloon-type protrusion upon further electron beam exposure (Fig. 3B).

### Vaporization and bubble creation of fluorescent microparticles

**Figure 4:**
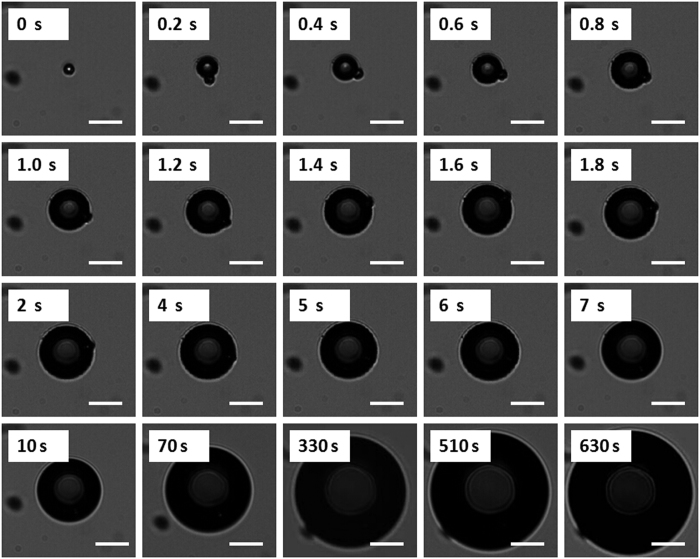
The sequence shows a 7.5 μm PFC liquid microparticle expanding and the newly bubble emerging over time upon laser irradiation, over the first 630 s. Scale bars represent 20 μm.

The vaporization and bubble evolution of fluorescent microparticles upon laser irradiation was observed in optical brightfield mode over time (Fig. 4). Brightfield images show that microparticles were vaporized completely in less than 100 ms. For one sample microparticle, an initial rapid increase in diameter from 7.5 μm to 37.1 μm was measured optically 1 s after vaporization, resulting in a radial expansion ratio of almost 5. After this initial rapid expansion, the bubble diameter slowly increased over time. Almost 11 min after vaporization, the bubble diameter had increased 13 - fold.

**Figure 5:**
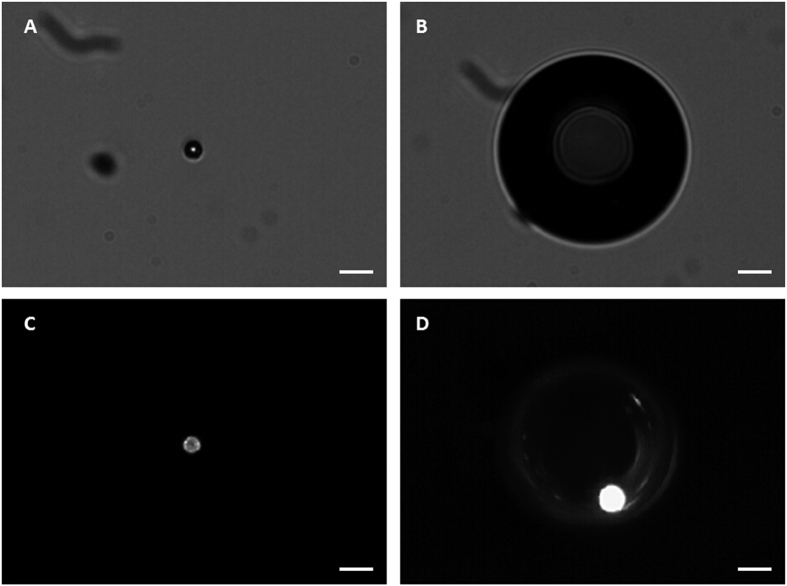
Brightfield and fluorescent images of a fluorescent microparticle before vaporization and the newly created bubble after vaporization. (**A**) Brightfield image of a microparticle before vaporization; (**B**) Brightfield image of the newly created bubble, which was taken approximately 11 s after vaporization; (**C**) Fluorescent image of a microparticle before vaporization; (**D**) Fluorescent image taken approximately 20 s after the brightfield image, which shows that after vaporization the fluorescence signal is localized to the interface between the new bubble and the original microparticle, with no apparent fluorescence visible within the newly created bubble. Scale bars represent 20 μm.

Figure 5 contains images of a fluorescent microparticle before vaporization and after vaporization, in which the newly created bubble can be clearly seen, both in brightfield and fluorescence modes. The brightfield image of the newly created bubble was taken approximately 11 s after vaporization (Fig. 5B). The fluorescence image was taken approximately 20 s after the brightfield image (Fig. 5D). Post-vaporization images were deliberately taken slightly off-focus, in order to depict the fluorescence pattern on the surface of the bubble. After vaporization, the fluorescence signal was localized to the interface between the new bubble and the original microparticle, with no apparent fluorescence visible within the newly created bubble.

**Figure 6:**
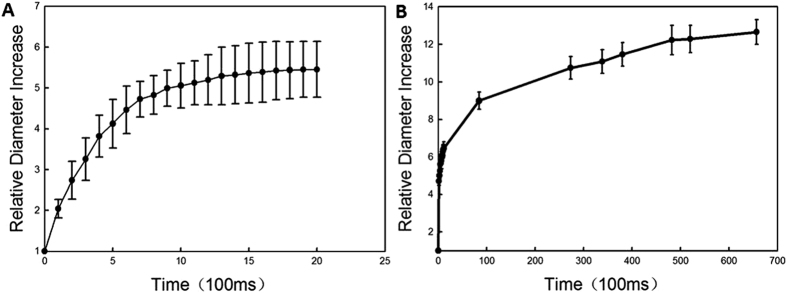
Increase in bubble diameter relative to the initial microparticle size as a function of time after vaporization in the first 2 s and 11 min after vaporization. (**A**) Bubble size change over the first 2 s; (**B**) Bubble size change over the first 11 min. The lines represent the mean ± standard deviation measurements of bubble size change, where 5 microparticles were monitored.

Bubble diameter growth from the initial pre-vaporized state the vaporized form, ranging from 5–8 μm in diameter, is graphically illustrated in Fig. 6. Within the first 2 s, the bubbles expanded to 4–6 times their original size (Fig. 6A). After 11 min, the bubbles were approximately 11–13 times their original size (Fig. 6B). The bubbles did not undergo rapid dissolution, at least within the first 11 min of observation.

### Microparticles in 2-D tumor cells

**Figure 7:**
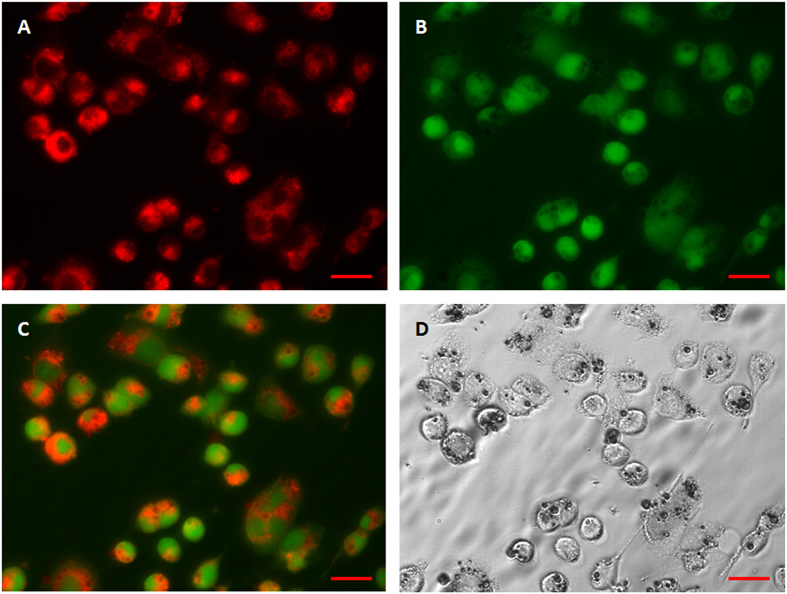
Fluorescent microscopy images of the MCF-7 cellular uptake of DiI-labelled microparticles and their intracellular distribution. (**A**) Under 549 nm laser excitation, red fluorescence represents microparticles; (**B**) Under 488 nm laser excitation, green fluorescence represents cells; (**C**) Merged picture of (**A**,**B)**; (**D**) Brightfield image. After incubation with DiI-labelled microparticles for 4 h, many microparticles had been phagocytized by the cells. Scale bars represent 20 μm.

To image the cellular uptake of DiI-labelled fluorescent microparticles, a fluorescent microscopic study was conducted. The fluorescent microparticles were incubated with FITC labelled MCF-7 cells. Under 549 nm and 488 nm laser excitations, strong red fluorescence at 549 nm (DiI) and green fluorescence at 488 nm (FITC) were observed, respectively. The results show that the microparticles were internalized by the cells and accumulated in the cytoplasm (Fig. 7).

**Figure 8:**
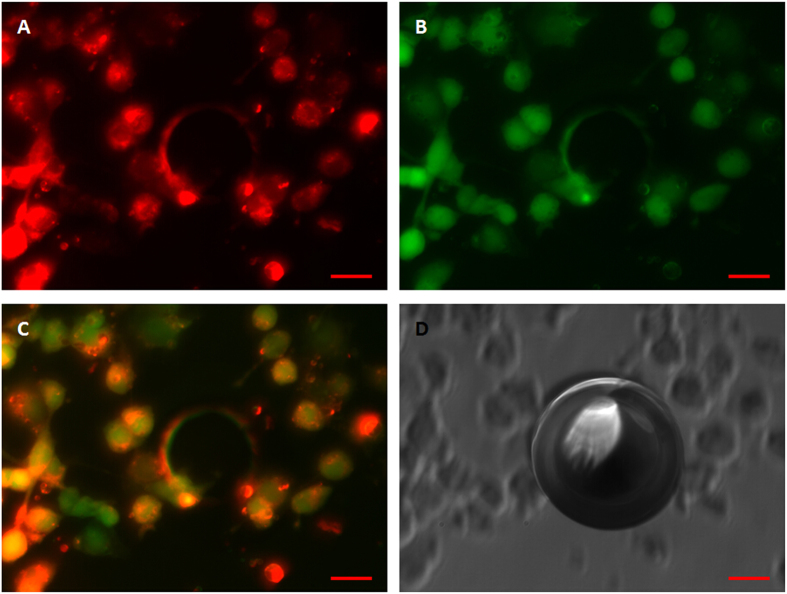
Fluorescent and brightfield micrographs of laser-vaporized DiI-labelled microparticles encapsulated in MCF-7 cells. (**A**) Under 549 nm laser excitation, red fluorescence represents microparticles; (**B**) Under 488 nm laser excitation, green fluorescence represents cells; (**C**) Merged picture of (**A**,**B**). (**D**) Brightfield image. Scale bars represent 20 μm.

To further verify that fluorescent microparticles could be vaporized within cells, a fluorescent microscopic study was conducted in both brightfield and fluorescence modes. Figure 8 shows fluorescence microscopy micrographs of MCF-7 cells phagocytized with post-vaporization DiI-labelled fluorescent microparticles. After vaporization, there was no discernable fluorescence signal within the newly created bubbles, while other microparticles that were not subjected to laser irradiation showed strong red fluorescence.

These results indicate that the DiI-labelled fluorescent microparticles could be efficiently phagocytized by tumor cells and vaporized upon laser irradiation. This demonstrates that microparticles hold much promise as delivery and release vehicles for drugs or genes through their vaporization within target cells.

### Microparticles in 3-D tumor spheroids

**Figure 9:**
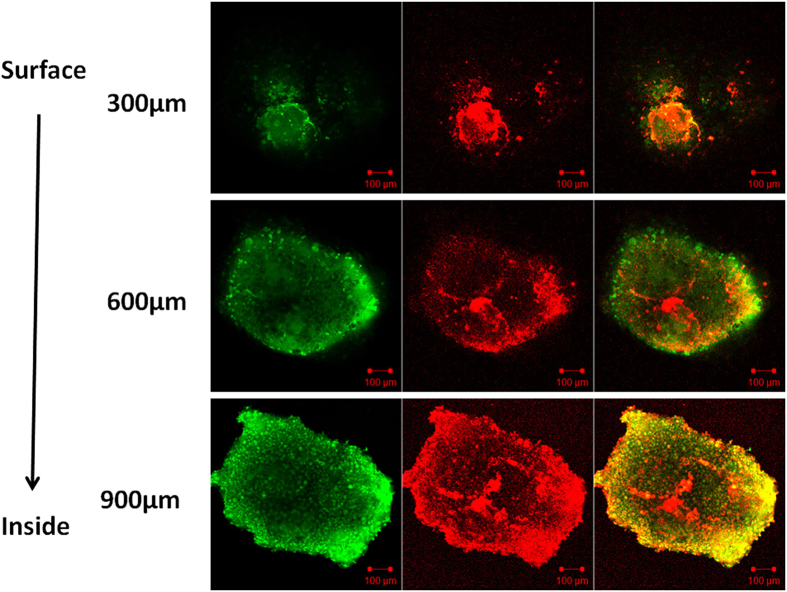
Confocal micrographs of the MCF-7 spheroid uptake of DiI-labelled 3-D microparticles and their intracellular distribution. The green fluorescence on the left represents cells under 488 nm laser excitation, the red fluorescence in the middle represents microparticles under 549 nm laser excitation; the green and red fluorescence on the right depicts the merged images of both the red and green fluorescence. After incubation with DiI-labelled microparticles for 7 days, 2-D tumor cells successfully formed 3-D spheroids. The green fluorescence labelled the cytoplasm and the red fluorescence labelled the microparticles, which accumulated in the cytoplasm. The distribution of the microparticles was analyzed by confocal microscopy using Z-stack imaging with 300 μm intervals. Scale bars represent 100 μm.

Following the described protocol, MCF-7 cells formed large (>800 μm) viable spheroids within 7 days of culture. Using the same method, spheroids incubated with fluorescent microparticles were also prepared. There was no significant difference in the spheroid size observed when the cells were grown with or without the microparticles. The cytoplasms of the MCF-7 cells were labelled with FITC and the fluorescent microparticles were labelled with DiI for confocal imaging. Under 549 nm and 488 nm laser excitations, strong red fluorescence at 549 nm (DiI) and green fluorescence at 488 nm (FITC) were observed, respectively. Confocal images showed that 2-D tumor cells formed 3-D spheroids successfully, where green fluorescence highlighted the cytoplasm and red fluorescence highlighted the microparticles, which accumulated in the cytoplasms (Fig. 9). These results indicate that microparticles can be internalized by cells without disturbing the formation of spheroids and with low toxicity.

**Figure 10:**
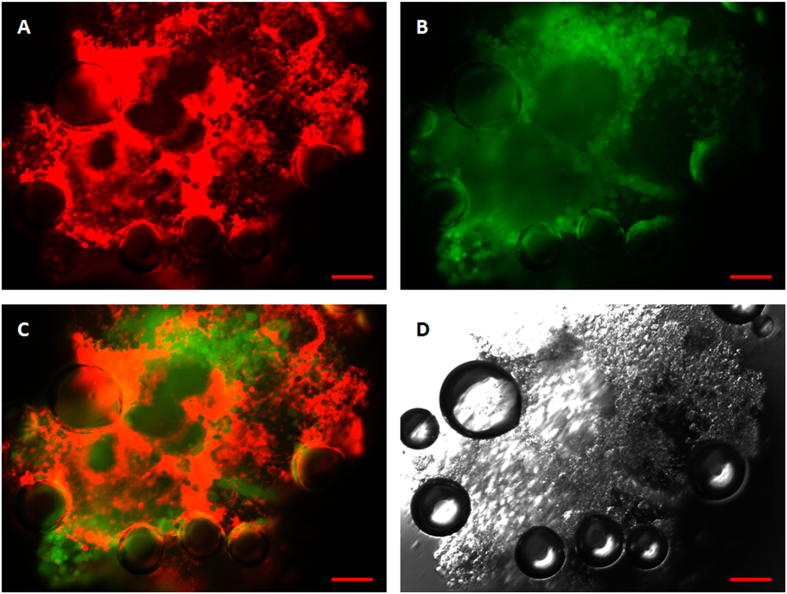
Fluorescent and brightfield micrographs of MCF-7 spheroid-encapsulated, laser-vaporized DiI-labelled microparticles. (**A**) Under 549 nm laser excitation, red fluorescence represents microparticles; (**B**) Under 488 nm laser excitation, green fluorescence represents cells; (**C**) Merged image of (**A**,**B)**. (**D**) Brightfield image. Scale bars represent 50 μm.

Fluorescence microscopy was also conducted to verify that fluorescent microparticles could be vaporized within the spheroids. Figure 10 shows post-vaporization DiI-labelled fluorescent microparticles vaporized by laser irradiation. The microparticles expanded to several times their original dimensions with no apparent fluorescence visible, in contrast to the microparticles with no expansion that exhibited strong red fluorescence.

### Cell cytotoxicity

**Figure 11:**
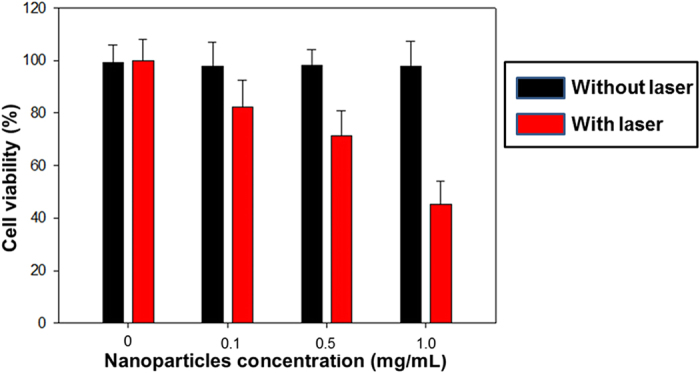
Cells viabilities of DiI-labelled microparticles on breast cancer cell viability with or without 532-nm laser irradiation.

To assess microparticle cytotoxicity, MCF-7 cells were incubated with DiI-labelled microparticles at concentrations of 0.1, 0.5 and 1.0 mg/mL for 4 h in 96-well plates. Then, a selection of the wells were exposed to laser irradiation (λ = 532 nm, 1.5 W/cm^2^) for 10 min, while an equal numble of wells were not exposed to irradiation. Cells without microparticles, irradiated and unirradiated, were used as the controls. After, all cell groups were further incubated for another 1 h and an MTT assay was conducted, the results of which are detailed in Fig. 11. Cell viability after laser irradiation was 82.3% ± 10.2%, 71.4% ± 9.5% and 45.3% ± 8.8%, for wells containing 0.1, 0.5 and 1.0 mg/mL microparticles, respectively. The cell viabilities recorded for the controls and the unirradiated cells, regardless of the microparticle concentration, showed no significant differences.

## Discussion

In the reported study, the laser initiated process of particle vaporization and the bubble stability following excitation in 2-D and 3-D cell models was investigated. Although PFC encapsulated particles have been explored extensively, previous studies were primarily focused on acoustic activation instead of optical activation. The results detailed above demonstrate that it is possible to encapsulate PFC within microparticles, which can then be vaporized into gas bubbles via ODV with absorbing materials encapsulated. As PFC is neither inherently hydrophobic or lipophobic, we employed PLGA as the outer shell to wrap the lipophilic fluorescent dye on the surface and trap the PFC in the core. PFH is a PFC with a boiling point of 56 °C; however, the boiling point is a little higher, closer to the thermal ablation temperature of tissue, when encapsulated within the particles^36^. We speculate that the fluorescent agent wrapped in the particles absorbed the laser and produced strong heating effects instantaneously, which caused the internal temperature in the microparticles to rise much higher than the boiling point of liquid PFH and ultimately induced the liquid-gas phase transition.

We observed the location of the fluorescent dye on the PLGA particles through fluorescence and confocal microscopy imaging. The homogeneous labelling of the PLGA shell was confirmed by both fluorescence microscopy and confocal microscopy. Figure 2 shows that the fluorescence marker selectively labelled the PLGA shell of the microparticles avoiding the PFC liquid cores, which emit no fluorescence signals. Following microparticle vaporization, which occurred in the first 100 ms of laser irradiation, the fluorescence signal only existed on the edge of the PLGA shell, while no fluorescence was discernable within the newly created PFH bubble (Fig. 2). Furthermore, following microparticle excitation, the fluorescence marker was rapidly expelled from the gas core of the bubble to the surface in between the bubbles and their surroundings, and was retained at the gas-liquid interface due to its hydrophobic nature. This result is consistent with a previous study concerning albumin shelled droplets by Reznik *et al*.^37^. Notably, it has been previously shown that microparticles can be used as drug carriers and have the ability to deliver a drug ‘payload’ upon vaporization^1^,^38^.

To the best of our knowledge, there is no report about the vaporization of microparticles under SEM scanning with high power electron beam irradiation. However, we present the first clear observation of vaporizing microparticles under SEM scanning. Vaporization on one side of the particle, similar to a balloon extruding to the outside, was clearly observed.

Figure 4 shows the vaporization of fluorescent microparticles through ODV and tracks the newly created bubble on optical brightfield-mode microscopy over time. The brightfield images show that the microparticles were vaporized completely in less than 100 ms. Vaporization via ADV is typically completed within hundreds of microseconds, as demonstrated in other published reports using an ultra-high speed camera^39^,^40^,^41^, which is much faster than the result reported here using ODV. However, Wei *et al*.^42^ reported on nanoemulsions comprised of PFC cores and gold nanospheres and demonstrated that vaporization was induced through ODV much faster than reported in out system. They observed that the initial bubble formation appeared in the first 200–300 ns, recorded using a coupled high speed camera with a frame speed of up to 2 × 10^8^ frame/s. However, the lifetime of the generated bubbles was short-lived, and they were lost after approximately 1400 ns. With simultaneous ultrasound exposure, the lifetime stability of the short-lived bubbles could be extended to more than 5 μs, Therefore, those bubbles were considerably less stable than the bubbles created in this study, which had lifetimes exceeding 10 min.

Bubble creation and subsequent expansion following vaporization were recorded. This expansion occurs due to the transport of gas from the surrounding bulk liquid into the bubble, which has been studied and reported on by Bull *et al*.^40^,^43^.

Bubble growth images and curves (Figs 4) indicate that the bubbles did not exhibit any signs of dissolution, at least within the first 11 min monitored in this study. This can be attributed, in part, to the low diffusivity and low solubility of PFH in water. Furthermore, the bubbles were comprised of a PLGA shell, which is more stable than the familiar microbubbles composed of lighter sulfur-hexafluoride (Sonovue) or perfluoropropane (Definity) gas^11^,^44^. Within the first 1 s of vaporization, the radial expansion ratio of our particle was almost 5x, which is close to the results reported for silica-coated lead sulfide nanoparticles loaded in PFC droplets vaporized via ODV (approximately 4.3 times the original droplet diameter)^9^ and micron-sized lipid-encapsulated decafluorobutane droplets vaporized via ADV (approximately 5 to 6 times the original droplet diameter)^45^. In this study, as shown in Fig. 6, the average ratio of the original particle surface area to the post-vaporization surface area was approximately 13 after 11 min. These larger type bubbles could be proposed for a number of applications, such as vessel occlusion^12^,^14^,^40^ and drug delivery^17^,^38^. It is important to note that the initial average size of our particles was in the micron-range, which may hinder their passage through blood vessels. Therefore, an aspect of our future work will be dedicated to the preparation of sub-micron scale particles that can be similarly vaporized and expanded.

The cellular uptake of DiI-labelled fluorescent microparticles and subsequent laser-induced vaporization was observed through optical and fluorescent microscopy. The results confirmed that even when the particles had been internalized within the cells, they could be successfully vaporized (Figs 8 and 9). Several reports have described how the process of vaporization and bubble expansion can destroy critical cell structures and create a reduction in tumor growth rates without any drug deployment^6^,^40^. Future work will also focus on the encapsulation of a therapeutic drug within the particles, with a view to delivering to target tissue delivery and ultimately drug release via vaporization for anti-tumor therapeutics.

The results detailed in Fig. 11, show that the microparticles themselves, without laser irradiation, show negligible cytotoxicity against cells. Moreover, exposure to laser irradiation in the absence of microparticles, had no adverse effect on viability. Contrastingly, post-irradiation, the cell viability decreased with increasing microparticle concentration. These results strongly indicate that the microparticles became cytotoxic after being vaporized through laser irradiation, resulting in cancer cell mortality, which is consistent with results reported by other investigators^46^,^47^,^48^.

In this work, we used a modified method to prepare spheroids with microparticles entrapped within which, to the best of the authors’ knowledge, has not been previously reported. The spheroids, which were large in size, were composed of cells co-cultured with particles for 7 days. The results indicate that the prepared microparticles exhibited little or no toxicity and did not inhibit spheroid growth. Confocal microscopy images demonstrate that 2-D tumor cells formed 3-D spheroids successfully, with green fluorescently labelled cytoplasm interspersed with red fluorescently labelled microparticles, which were phagocytized by the cells of the spheroids (Fig. 9). Furthermore, the results highlighted in Fig. 10 confirm that the spheroid encapsulated particles could be vaporized by laser irradiation. Planned future work includes the incorporation of an encapsulated drug, which will be used to evaluate the penetration depth and the anti-tumor effects of this approach in different spheroidal zones, such as proliferation, quiescent or necrotic zones.

In conclusion, the results of this study demonstrate the theranostic agent potential of the novel phase-shift PLGA particles. Our experiments demonstrate that the phase-shift agent provides a feasible method for drug delivery and thermal ablation to targeted tissues and organs *in vivo*.

## Materials

### Fluorescent shell PFC microparticle preparation

Fluorescent shell PFC microparticles were prepared as follows: DiI, a lipophilic fluorescent marker (Molecular Probes Inc., Eugene, OR), was dissolved in 2 mL methylene chloride and mixed with an organic solution of 100 mg PLGA (lactide: glycolide = 50:50, MW = 10,000 Da; Shandong Key Laboratory of Medical Polymer Materials, China) polymer by stirring. Then, 100 μL PFH (Fluoromed, Round Rock, TX, USA) was added to the organic phase, and the mixture was emulsified using a sonicator (Sonics & Materials, Inc., Newtown, USA) for 30 s. Subsequently, 10 mL of 5% cold Poly (vinyl alcohol) (PVA, MW = 30,000–70,000 Da; Sigma-Aldrich, USA) solution was added to the initial emulsion, and the mixture was homogenized using a tissue-tearor (Biospec, USA) for 5 min at 9,500 rpm. The suspension was then poured into 20 mL of a 2% isopropanol alcohol solution and stirred at room temperature for 1 h to evaporate the redundant methylene chloride. The resulting double emulsion was left to settle for 1 h to allow the microparticles to sink to the bottom of the container. The supernatant was discarded and the precipitate was resuspended in distilled water. The resuspension procedure was repeated three times in order to minimize the background fluorescence signal from the surrounding liquid.

### Cell culture and breast cancer cell spheroid formation

The human breast cancer cell line MCF-7 was obtained from American Type Culture Collection (ATCC) and cultured as recommended. To initiate spheroid formation, matrigel solution was prepared using 2.5% w/v matrigel in phosphate buffered saline (PBS) solution. MCF-7 cells were trypsinized from their culture flasks and 10^7^ cells were mixed with 200 μL culture medium supplemented with 5 μL matrigel solution. The cell suspension was seeded onto the cover of a 96-well plate (5 × 10^5^ cells per well with a view to establishing a hanging culture), 100 μL of culture medium was added to each well, and the plates were incubated under 37 °C, 5% CO_2_, and 95% relative humidity conditions. Spheroid formation and growth (800–900 μm diameters) were monitored using an Olympus IX 81 fluorescent microscope. To investigate if the microparticles can be incorporated into the spheroids, the cells were prepared as described, with the addition of fluorescent microparticles, and incubated for 7 days. The forming spheroids were monitored using confocal microscopy.

### Optical and fluorescent microscopy

Fluorescent shell PFC microparticles were placed on glass slides and observed with an Olympus IX81 inverted optical microscope (Olympus, Japan). Fluorescent microparticles dyed with DiI were excited at 549 nm and observed at 565 nm. Cell cytoplasm was labelled with fluorescein isothiocyanate (FITC) and excited at 488 nm and observed at 525 nm.

### Confocal microscopy

In order to verify the location of the fluorescent marker, the prepared microparticle suspensions were examined with a Zeiss LSM-510 confocal scanning laser microscope (LSM 510, Carl Zeiss, Canada) with a 63x objective (NA 1.40, oil immersion). Red fluorescence was observed with a long-pass 565 nm emission filter under 549 nm laser illumination. FITC was used to stain the cytoplasm of MCF-7 cells and spheroids (diameter = 800–900 μm) to show cell location and to facilitate the definition of spheroid boundaries by microscopic examination.

### Scanning electron microscopy

The morphological features of the microparticles were observed through SEM after being dried in air (S-3400N, Hitachi, Japan). Sample suspensions were sputtered with gold to make them conductive, dried at room temperature, and deposited on a copper stub before imaging.

### Laser irradiation optical microscopy

**Figure 12:**
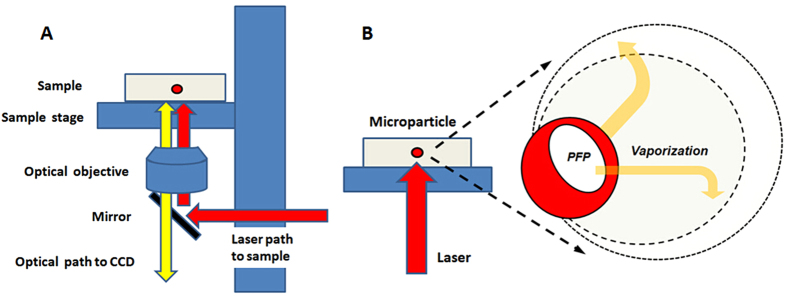
(**A**) Experimental setup used for optical observation of microparticle vaporization. The laser was focused through the platform onto the sample. Wavelengths of 500–650 nm were reflected by the mirror toward the sample, but other wavelengths were also allowed to pass for optical viewing. (**B**) Schematic depicting the vaporization of a single microparticle under laser irradiation.

The experimental apparatus used for the optical observation of microparticle vaporization is shown in Fig. 12. An Olympus IX81 inverted optical microscope connected to a 532 nm laser (Teem Photonics, France) and a high-speed camera (Fast Cam APX RS, Photron, San Diego, CA, USA) was used for microparticle vaporization and optical and fluorescent imaging. For microparticle vaporization, the laser was focused on a chamber slide (Nunc, Germany) containing a highly diluted microparticle sample in distilled water. This ensured that the microparticles were sufficiently far apart from each other to probe them individually. Ten microparticles were measured with the laser focused on each individual microparticle each time.

The laser was focused to a 4 μm spot size using a 10x objective with a 0.3 numerical aperture, giving a laser fluence ranging from 0.05 J/cm^2^ to 3.4 J/cm^2^ per pulse. As the laser fluence was increased, the process was repeated until vaporization occurred or the maximum laser fluence level was reached.

For studies involving cell encapsulated microparticles or spheroid vaporization, the cells or spheroids were placed in a chamber slide filled with culture medium and maintained at 37 °C. The laser ablation procedure was the same as for the individual microparticles.

### *In vitro* cytotoxicity assay

The cytotoxicity of DiI-labelled microparticles was evaluated through MTT assays with the MCF-7 cell line. Briefly, various concentrations of DiI-labelled microparticles in medium were incubated with cells for 4 hours at 37 °C. Then, for all microparticle concentrations, a selection of the wells were exposed to laser irradiation (λ = 532 nm, 1.5 W/cm^2^) for 10 min, while an equal number of wells were not exposed to irradiation. Cells without microparticles, irradiated and unirradiated, were used as the controls. After, all cell groups were further incubated for another 1 h. Then MTT solution (20 μL, 5 mg/mL) was added to each well to replace the medium for a further 4 hours incubation at 37 °C. The formazan crystals were dissolved with DMSO (100 μL) for 15 min and the absorbance was measured by a microplate reader (Bio-Rad Laboratories, CA) at a wavelength of 490 nm. The values are reported as the mean ± SD (n = 3).

## Additional Information

**How to cite this article**: Niu, C. *et al*. Laser irradiated fluorescent perfluorocarbon microparticles in 2-D and 3-D breast cancer cell models. *Sci. Rep.* **7**, 43408; doi: 10.1038/srep43408 (2017).

**Publisher's note:** Springer Nature remains neutral with regard to jurisdictional claims in published maps and institutional affiliations.
